# Two Sides to Every Question: Attempts to Activate Chicken Innate Immunity in 2D and 3D Hepatic Cell Cultures

**DOI:** 10.3390/cells10081910

**Published:** 2021-07-27

**Authors:** Csilla Sebők, Patrik Tráj, Júlia Vörösházi, Máté Mackei, Márton Papp, Péter Gálfi, Zsuzsanna Neogrády, Gábor Mátis

**Affiliations:** 1 Division of Biochemistry, Department of Physiology and Biochemistry, University of Veterinary Medicine, István utca 2., H-1078 Budapest, Hungary; traj.patrik@univet.hu (P.T.); voroshazi.julia@univet.hu (J.V.); mackei.mate@univet.hu (M.M.); neogrady.zsuzsanna@univet.hu (Z.N.); matis.gabor@univet.hu (G.M.); 2 Centre for Bioinformatics, University of Veterinary Medicine, István utca 2., H-1078 Budapest, Hungary; papp.marton@univet.hu; 3 Department of Pharmacology and Toxicology, University of Veterinary Medicine, István utca 2., H-1078 Budapest, Hungary; galfi.peter@univet.hu

**Keywords:** hepatocyte, chicken, liver, inflammation, spheroid, co-culture, interleukin

## Abstract

The liver with resident tissue macrophages is the site of vivid innate immunity, activated also by pathogen-associated molecular patterns (PAMPs) leaking through the intestinal barrier. As gut-derived inflammatory diseases are of outstanding importance in broiler chickens, the present study aimed to establish a proper hepatic inflammatory model by comparing the action of different PAMPs from poultry pathogens on chicken 2D and 3D primary hepatocyte—non-parenchymal cell co-cultures, the latter newly developed with a magnetic bioprinting method. The cultures were challenged by the bacterial endotoxins lipopolysaccharide (LPS) from *Escherichia coli*, lipoteichoic acid (LTA) from *Staphylococcus aureus* and by enterotoxin (ETxB) from *Escherichia coli*, *Salmonella* Typhimurium derived flagellin, phorbol myristate acetate (PMA) as a model proinflammatory agent and polyinosinic polycytidylic acid (poly I:C) for mimicking viral RNA exposure. Cellular metabolic activity was assessed with the CCK-8 test, membrane damage was monitored with the lactate dehydrogenase (LDH) leakage assay and interleukin-6 and -8 (Il-6 and -8) concentrations were measured in cell culture medium with a chicken specific ELISA. Both LPS and LTA increased the metabolic activity of the 3D cultures, concomitantly decreasing the LDH leakage, while in 2D cultures ETxB stimulated, PMA and poly I:C depressed the metabolic activity. Based on the moderately increased extracellular LDH activity, LTA seemed to diminish cell membrane integrity in 2D and poly I:C in both cell culture models. The applied endotoxins remarkably reduced the IL-8 release of 3D cultured cells, suggesting the effective metabolic adaptation and the presumably initiated anti-inflammatory mechanisms of the 3D spheroids. Notwithstanding that the IL-6 and IL-8 production of 2D cells was mostly not influenced by the endotoxins used, only the higher LTA dose was capable to evoke an IL-8 surge. Flagellin, PMA and poly I:C exerted proinflammatory action in certain concentrations in both 2D and 3D cultures, reflected by the increased cellular IL-6 release. Based on these data, LTA, flagellin, PMA and poly I:C can be considered as potent candidates to induce inflammation in chicken primary hepatic cell cultures, while LPS failed to trigger proinflammatory cytokine production, suggesting the relatively high tolerance of avian liver cells to certain bacterial endotoxins. These results substantiate that the established 3D co-cultures seemed to be proper tools for testing potential proinflammatory molecules; however, the remarkable differences between 2D and 3D models should be addressed and further studied.

## 1. Introduction

The liver serves as the primary organ barrier for the gut-derived antigenic load and protects the systemic circulation against both residual oxidative and pathogen burden originated from the gastrointestinal tract. Broiler chickens with an immature immune system are prone to develop dysbacteriosis and necrotic enteritis. Disruption of the intestinal integrity would lead to leakage of microbial toxins and byproducts through the epithelial barrier leading to inflammation, remarkably repressing weight gain and thus productivity in poultry [1]. Modeling these pathologies *in vitro* is relevant to finding potential molecules for the prophylaxis of hepatic inflammatory and oxidative damage induced by gut-originated pathogen load. Currently, there is an urgent need to designate potential antibiotic alternatives to make the proper and responsible antibiotic application of veterinary medicine possible and diminish the extent of antibiotic resistance as the most crucial public health risk of the future [2].

Several studies about inflammatory molecules have been performed using two-dimensional (2D) *in vitro* cell cultures, albeit it has been stated that some 2D cultures are not entirely capable of reflecting physiological conditions [3]. Cells in 2D cultures tend to lose their natural polarization and differentiated phenotype because they connect less with each other and more with the surface of the cell culture plate [4]. It has been proved that hepatocytes cultured in 2D conditions lose their typical characteristics, but some of these properties remain intact in 3D spheroid cultures because primary hepatocytes can maintain their cuboidal geometry. Therefore they can stay at a relatively stable, differentiated condition [5]. The aspects mentioned above emphasize the application of a proper cellular model for a specific objective. Hence, we aimed to investigate the adaptability of 2D and 3D chicken hepatic co-cultures to simulate the hepatic inflammatory response.

Owing to the vast majority of resident tissue macrophages and the role of producing soluble and membrane-bound pathogen-recognition receptors, complement factors, and acute-phase proteins, the liver conducts innate immunity [6,7]. Toll-like receptors (TLR) present on each Kupffer cell, hepatocyte, biliary epithelial cell and sinusoidal endothelial cell are subject to microbial pathogen-associated molecular patterns (PAMP) of portal origin. Similarly to mammals, chicken expresses all of the indispensable TLRs apart from TLR-8 and -9. Signal transduction triggered by TLR agonists ensues downstream of adaptor molecules, resulting in the production of a wide range of proinflammatory mediators [8,9,10].

Several types of PAMPs may be applied to trigger hepatic cellular inflammatory and stress response *in vitro*. The TLR agonists of the highest importance and clinical relevance are lipopolysaccharides (LPS) derived from the Gram-negative bacterial cell wall [11]. Along with its Gram-positive bacterial counterpart, lipoteichoic acid (LTA), it can either modulate signal transduction through TLR activation or bind aspecifically to type-I scavenger receptor in the liver [6,9]. LPS from O55:B5 chicken pathogen *Escherichia coli* is most frequently used *in vitro* on chicken cell lines and cultures] [10,12]. *Staphylococcus aureus* LTA proved to induce oxidative burst via TLR activation initiated protein kinase C (PKC) dependent transduction in chicken heterophil granulocytes [13].

Apart from the conventional cAMP-mediated pathomechanism of porcine post-weaning diarrhea triggered by heat-labile enterotoxin of *Escherichia coli*, there is much more to unfold about the effect of this toxin. Besides the chloride ion channel activation, a proinflammatory effect is triggered solely by the beta subunit of the molecule (ETxB), known previously as the non-functional membrane-binding domain. This pentamer molecule is hypothesized to act by the translocation of NF-kappa B to the nucleus of the cells [14].

Flagellin exerts an increase in cytokine gene expression and causes notable degranulation and oxidative burst in chicken heterophil granulocyte culture [15,16]. The presence of free bacterial flagellin in the living organism is hypothesized to result from the disintegration or the leaky assembly of the organ of bacterial locomotion, the flagellum. Foreseeable that the highly conserved hidden core regions of this motor protein induce the activation of the non-specific immune system considerably via a TL receptor, TLR-5 [17].

Phorbol myristate acetate (PMA) is commonly used in human medical research both in vivo and *in vitro* to induce inflammation and thus challenge the therapeutic effects of substances with anti-inflammatory nature. Applying PMA was coupled to elevated proinflammatory cytokine release (IFN-gamma, TNF-alpha, IL-6) and COX-2 expression [18,19]. Meanwhile, the oxidative response induced by PMA is remarkably stronger than the ones triggered by LTA and LPS stimulation; a selective PKC inhibitor could block this activation. Therefore, it presumably activates the cellular inflammatory response in a somewhat different manner [20].

Polyinosinic polycytidylic acid (poly I:C) as an interferon inducer is another novel alternative to evoke inflammation in cell cultures. These molecules show a similar structure to viral double-stranded RNA genome characteristic for *Reoviridae* and *Birnaviridae* virus families with abundant poultry specific strains. The molecule exerts inflammatory cytokine synthesis via TLR-3 receptors expressed on the endosomal surface both in mammalian and avian cells following the anticipated internalization of the macromolecular structure [21,22].

The primary aim of the present study was to compare the putative proinflammatory action of certain PAMPs on 2D and 3D hepatic cell co-cultures of chicken origin. In the first study, the effect of two bacterial endotoxins was investigated, specifically LPS from *Escherichia coli* and LTA from *Staphylococcus* *aureus*. In the second study, we analyzed further PAMPs potentially triggering inflammation, such as enterotoxin of *Escherichia coli*, flagellin from *Salmonella* Typhimurium, PMA and poly I:C. By screening a wide range of potential proinflammatory substances, these results would serve as fundamental data to set and characterize an inflammatory chicken hepatic cell model suitable for challenging substances with antioxidant and immunomodulatory effects.

## 2. Materials and Methods

The animals were reared and fed according to the Ross technology [23]. Cell isolation was performed in strict accordance with the international and national law along with institutional guidelines and was confirmed by the Local Animal Welfare Committee of the University of Veterinary Medicine, Budapest and by the Government Office of Zala County, Food Chain Safety, Plant Protection, and Soil Conservation Directorate, Zalaegerszeg, Hungary (number of permission: GK-419/2020; approval date: 11 May 2020).

### 2.1. Cell Isolation and Culturing

Liver cells were freshly isolated from three-week-old Ross-308 male broiler chickens (obtained from Gallus Poultry Farming and Hatching Ltd., Devecser, Hungary), as Mackei et al. [24] described. All chemicals used for cell isolation and culturing were purchased from Merck KGaA (Darmstadt, Germany) except when otherwise specified.

The animals were decapitated in CO_2_ narcosis and the liver was perfused via the gastropancreaticoduodenal vein of the hepatic portal system with different buffer solutions. During the multistep perfusion, all buffers were warmed up to 40 °C and were freshly oxygenated with Carbogen (95% O_2_, 5% CO_2_). The velocity of the perfusion was 30 mL/min. First, 150 mL Hanks’ Balanced Salt Solution (HBSS) buffer containing 0.5 M ethylene glycol tetraacetic acid (EGTA) was applied, followed by 150 mL EGTA-free HBSS. Lastly, the liver was perfused by 100 mL HBSS buffer freshly supplemented with 100 mg collagenase type IV (Nordmark, Uetersen, Germany), 7 mM CaCl_2_ and 7 mM MgCl_2_. The collagenase-mediated digestion of the liver tissue was applied in order to disintegrate the hepatic cells. After excision and disruption of the Glisson’s capsule, the freshly gained cells were suspended in 50 mL ice-cold HBSS buffer containing bovine serum albumin (BSA, 2.5%) to avoid cluster formation, filtered through three layers of sterile gauze to remove any leftover cell aggregates and the undigested interstitium, then incubated on ice for 50 min. Thereafter, the hepatocyte and non-parenchymal cell containing fractions were separated using multistep differential centrifugation. The cell suspension was centrifuged three times at 100× *g* for 3 min in Williams’ Medium E previously supplemented with 0.22% NaHCO_3_, 50 mg/mL gentamycin, 2 mM glutamine, 4 µg/L dexamethasone, 20 IU/L insulin, 0.5 µg/mL amphotericin-B and 5% fetal bovine serum (FBS). After each step, non-parenchymal cells containing supernatants were collected separately and hepatocyte containing pellets were freshly resuspended in the cell culture medium. Eventually, a purified hepatocyte fraction was received.

In order to separate the non-parenchymal cell fraction, the supernatant was centrifuged at 350× *g* for 10 min to sediment the remaining hepatocytes and red blood cells. After this, the supernatant was centrifuged again at 800× *g* for 10 min. The suspension gained after resuspending the final sediment contained the non-parenchymal cell enriched fraction. The viability of the cells was examined by trypan blue exclusion test, the number of viable cells should be over 90%. The cell load was determined by cell counting in Bürker’s chamber to adjust the appropriate cell concentrations (10^6^ cells/mL for 2D cultures and 5 × 10^5^ cells/mL for 3D cultures). Both hepatocyte and non-parenchymal cell enriched fractions have been previously characterized by flow cytometry and immunofluorescent detection of specific markers for hepatocytes and macrophages [24].

#### 2.1.1. 2D Cell Cultures

**Figure 1:**
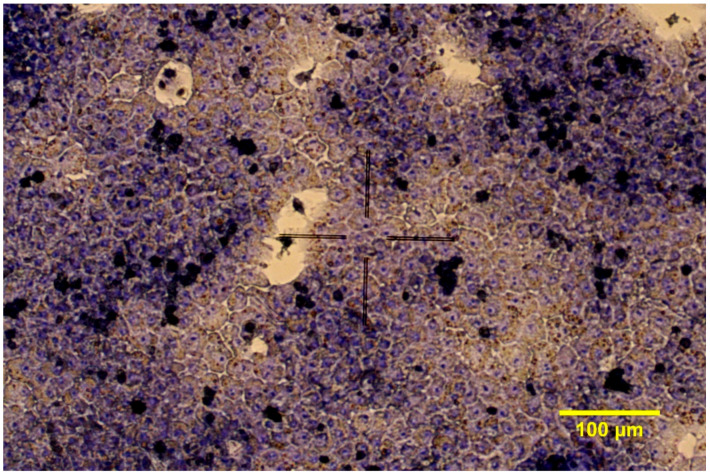
Giemsa staining of chicken hepatocyte—non-parenchymal cell co-cultures in 2D conditions (200× magnification).

After mixing the cell suspensions in the ratio of 6:1 (hepatocytes to non-parenchymal cells), the hepatocyte- non-parenchymal cell co-cultures were seeded onto 96-well plates (Greiner Bio-One Hungary Kft., Mosonmagyaróvár, Hungary) previously coated with collagen type I (10 μg/cm^2^). The seeding volume was 100 µL/well. The 2D cell cultures were incubated at 37 °C in humid atmosphere with 5% CO_2_. Culture media were changed after 4 h and confluent co-cultures were gained after 24 h (Figure 1). Culture medium contained 5% FBS only in the first 24 h of culturing. Other supplements added to the medium during the experiment were the same as in the Williams’ Medium E used for the seeding, namely 0.22% NaHCO_3_, 50 mg/mL gentamycin, 2 mM glutamine, 4 µg/L dexamethasone, 20 IU/L insulin, 0.5 µg/mL amphotericin-B.

#### 2.1.2. 3D Cell Cultures

All equipment and chemicals needed for the 3D cell culturing were purchased from Greiner Bio-One Hungary Kft., Mosonmagyaróvár, Hungary. To magnetize the cells, 500 µL magnetic nanoparticle (NanoShuttleTM-PL) was added to 5 mL co-culture suspension (previously mixing cell suspensions in the ratio of 6:1, hepatocyte to non-parenchymal cells). The cells were seeded onto 96-well cell repellent plates provided by the manufacturer. The seeding volume was 100 µL/well. The plates were incubated at 37 °C for 1 h to get the nanoparticles attached to the cell membrane. Thereafter, the plates were placed on a magnetic drive with magnets under each well (Spheroid Drive) and were incubated at 37 °C in humid atmosphere with 5% CO_2_.

**Figure 2:**
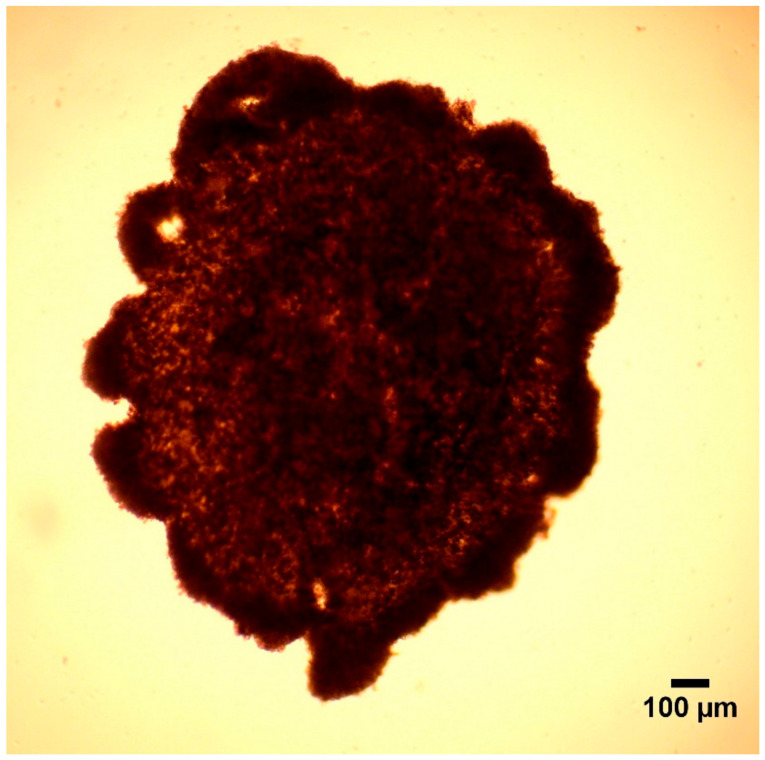
Hepatocyte—non-parenchymal cell co-culture in form of spheroid (40× magnification).

The culture media was changed after 24 h. For this, the plate was placed on a magnetic Holding Drive. Then, the 3D cell cultures were incubated for an additional 24 h on the Spheroid Drive. The plate was left on the Spheroid Drive altogether for 48 h to produce adequate spheroids (Figure 2). Culture medium (Williams’ Medium E) contained 5% FBS only in the first 24 h of culturing. All of the other supplements were provided during the entire experiment, which were 0.22% NaHCO_3_, 50 mg/mL gentamycin, 2 mM glutamine, 4 µg/L dexamethasone, 20 IU/L insulin, 0.5 µg/mL amphotericin-B.

### 2.2. Treatment of Cultured Cells

#### 2.2.1. Study 1

The medium of the 2D and 3D cell cultures was supplemented with 0 (control), 10 or 50 µg/mL LPS from *Escherichia coli* (O55:B5) for 24 h [13,15,25,26], further with 10 or 50 µg/mL LTA from *Staphylococcus aureus* [13].

#### 2.2.2. Study 2

Both 2D and 3D cell cultures were exposed to culture media supplemented with 0 (control), 20 or 50 µg/mL B subunit of the heat-labile enterotoxin derived from *Escherichia coli* (ETxB) [27,28], 100 or 250 ng/mL *Salmonella* Typhimurium derived flagellin [15,29,30], 100 or 1000 ng/mL phorbol myristate acetate (PMA) [20], further with 50 or 100 µg/mL polyinosinic polycytidylic acid (poly I:C) [29,30] for 24 h. To achieve the re-annealing the poly I:C was heated at 50 °C for 3 min then cooled down before added to cell culture media.

### 2.3. Measurements

The metabolic activity of the cells was measured on 96-well plates by CCK-8 assay (Cell counting Kit-8, Dojindo Molecular Technologies, Rockville, MD, USA), detecting the amount of NADH+H^+^ gained in the catabolic pathways (n_study 1_ = 6, n_study 2_ = 10). The test was performed according to the manufacturer’s protocol. First, 10 µL CCK-8 reagent and 100 µL fresh Williams’ Medium E were added to the cultured cells, and after a 2-h incubation, the absorbance was measured at 450 nm with a Multiskan GO 3.2 reader (Thermo Fisher Scientific, Waltham, MA, USA).

In order to investigate the rate of plasma membrane damage as a consequence of cell injury, lactate dehydrogenase (LDH) activity in the culture media was measured by an enzyme kinetic photometric assay (Diagnosticum Ltd., Budapest, Hungary). First, 200 µL working reagent (containing 56 mM phosphate buffer, pH 7.5; 1.6 mM pyruvate and 240 µM NADH+H^+^) was mixed with 10 µL cell culture medium. The absorbance was measured at 340 nm with a Multiskan GO 3.2 reader (n_study 1_ = 6, n_study 2_ = 5).

The concentrations of interleukin-6 (IL-6) and interleukin-8 (IL-8) were measured in the culture media by chicken specific ELISA kits (MyBioSource, San Diego, CA, USA) following the manufacturer’s protocol. The absorbance was measured at 450 nm with a Multiskan GO 3.2 reader (n_study 1_ = 6, n_study 2_ = 5).

### 2.4. Statistical Analysis

All statistical analysis was performed in R v. 4.0.3 (R Core Team, 2020). We calculated the LDH activity by measuring the absorbance six times, averaging the differences between the consecutive time points. For visualization, relative intensity (for the CCK results), relative concentration values (for interleukin measures), and relative change in absorbance (for the LDH activity) were calculated from the data by dividing each value by the average of the corresponding control group. Plots were generated using the ggplot2 package (Wickham, 2016). Statistical significance was evaluated for each treatment to the corresponding control group on the raw data, using Wilcoxon signed rank test. If the p-value was less than 0.05 we have considered a difference significant.

## 3. Results

### 3.1. Metabolic Activity

**Figure 3:**
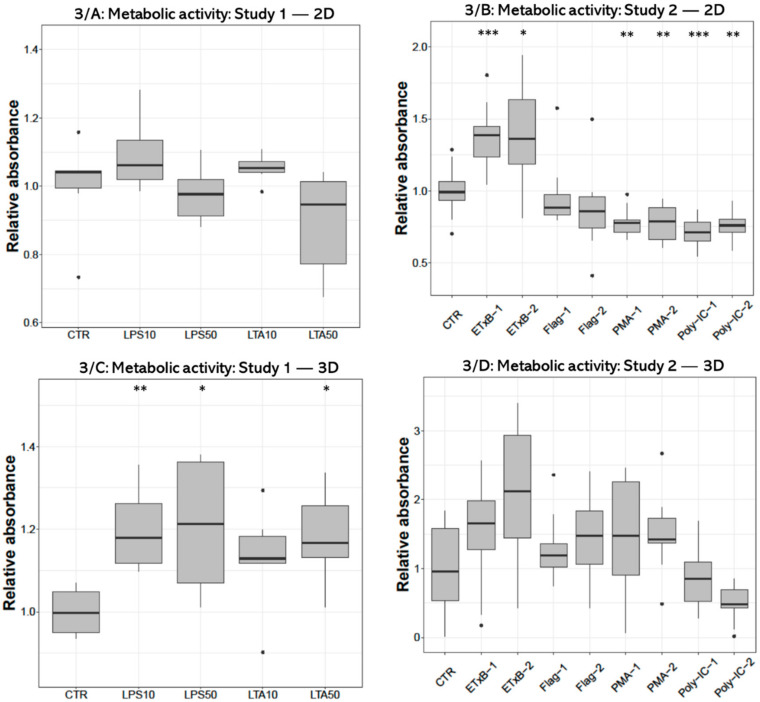
Boxplots showing the metabolic activity of hepatocyte—non-parenchymal cells in 2D (Study 1: (**A**) and Study 2: (**B**)) and 3D (Study 1: (**C**) and Study 2: (**D**)) co-cultures indicated by the CCK-8 assay (n_study 1_ = 6/group, n_study 2_ = 10/group). Relative absorbances were calculated by considering the mean value of control cultures as 1. The “CTR” refers to control cells that received none of the treatments. The treatments were: LPS10 and LPS50 = 10 and 50 µg/mL lipopolysaccharide (LPS) from *Escherichia coli*, LTA10 and LTA50 = 10 and 50 µg/mL lipoteichoic acid (LTA) from *Staphylococcus aureus*, ETxB-1 and -2 = 20 and 50 µg/mL subunit B of heat-labile enterotoxin of *Escherichia coli*, Flag-1 and -2 = 100 and 250 ng/mL flagellin from *Salmonella* Typhimurium, PMA-1 and -2 = 100 and 1000 ng/mL phorbol myristate acetate (PMA), poly-IC-1 and -2 = 50 and 100 µg/mL polyinosinic polycytidylic acid (poly I:C). Asterisks over the boxes refer to significant differences compared to “CTR” cells within the same cell culture model and the same study. * *p* < 0.05, ** *p* < 0.01, *** *p* < 0.001.

Metabolic activity measured with CCK-8 test after treatment with 10 and 50 µg/mL LPS and 50 µg/mL LTA concentration was significantly higher (*p* = 0.0050, *p* = 0.0301, *p* = 0.0200, respectively) in 3D-cultured cells compared to control (Figure 3C). No alteration was found in 2D cell cultures after LPS or LTA exposure (Figure 3A). There was a significant increase after 20 and 50 µg/mL enterotoxin treatment (*p* < 0.001, *p* = 0.0232, respectively), and a significant decrease after applying 100 and 1000 ng/mL PMA (*p* = 0.0041, *p* = 0.0041, respectively), as well as when using 50 and 100 µg/mL poly I:C (*p* < 0.001, *p* = 0.0041, respectively) in 2D-cultured cells (Figure 3B). No significant effect was detected in 3D cultures after the same treatments (Figure 3D).

### 3.2. LDH Activity

**Figure 4:**
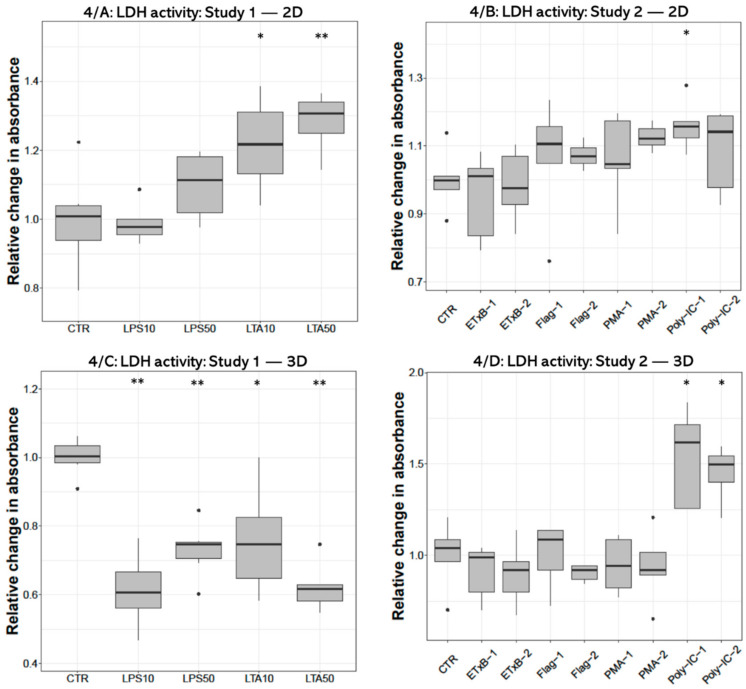
Boxplots showing the extracellular lactate dehydrogenase (LDH) activity of hepatocyte—non-parenchymal cells in 2D (Study 1: (**A**) and Study 2: (**B**)) and 3D (Study 1: (**C**) and Study 2: (**D**)) co-cultures as indicated by enzyme kinetic assay (n_study 1_ = 6/group, n_study 2_ = 5/group). Relative changes in absorbances were calculated by considering the mean value of control cultures as 1. The “CTR” refers to control cells that received none of the treatments. The treatments were: LPS10 and LPS50 = 10 and 50 µg/mL lipopolysaccharide (LPS) from *Escherichia coli*, LTA10 and LTA50 = 10 and 50 µg/mL lipoteichoic acid (LTA) from *Staphylococcus aureus*, ETxB-1 and -2 = 20 and 50 µg/mL subunit B of heat-labile enterotoxin of *Escherichia coli*, Flag-1 and -2 = 100 and 250 ng/mL flagellin from *Salmonella* Typhimurium, PMA-1 and -2 = 100 and 1000 ng/mL phorbol myristate acetate (PMA), poly-IC-1 and -2= 50 and 100 µg/mL polyinosinic polycytidylic acid (poly I:C). Asterisks over the boxes refer to significant differences compared to “CTR” cells within the same cell culture model and the same study. * *p* < 0.05, ** *p* < 0.01.

Concerning the extracellular LDH activity significant increase was found after both (10 and 50 µg/mL) LTA concentrations (*p* = 0.0260, *p* = 0.0043, respectively) in 2D cultures (Figure 4A). On the contrary, LDH activity was decreased following all LPS (*p* = 0.0022, *p* = 0.0050, respectively) and LTA treatments (*p* = 0.0161, *p* = 0.0050) in 3D-cultured cells (Figure 4C). Significant elevation was detected after 50 ng/mL poly I:C treatment (*p* = 0.0318) in 2D cultures (Figure 4C), and after both concentrations of poly I:C (*p* = 0.0119, *p* = 0.0160) in 3D cultures (Figure 4D).

### 3.3. IL-6 Concentration

**Figure 5:**
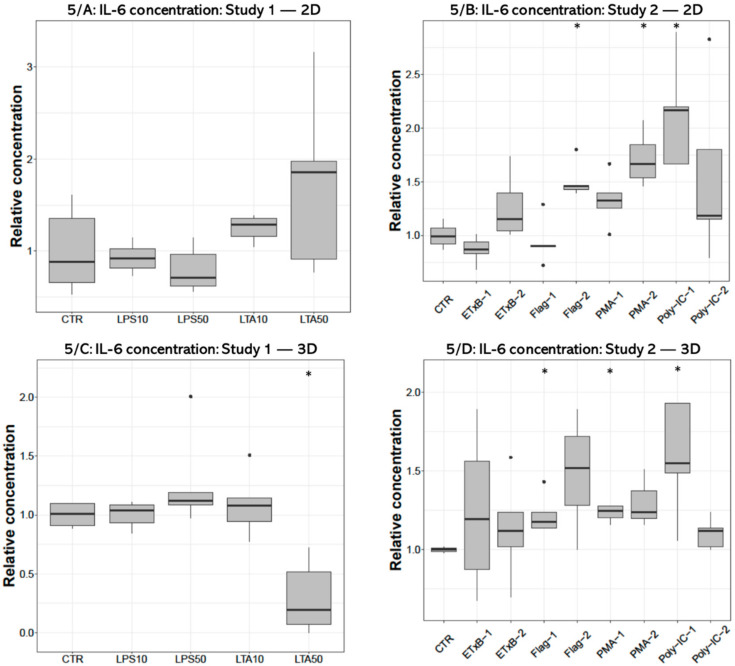
Boxplots showing the interleukin-6 (IL-6) concentration in cell culture media of hepatocyte—non-parenchymal cells in (Study 1: (**A**) and Study 2: (**B**)) and 3D (Study 1: (**C**) and Study 2: (**D**)) co-cultures detected by a chicken specific ELISA assay (n_study 1_ = 6/group, n_study 2_ = 5/group). Relative concentrations were calculated by considering the mean value of control cultures as 1. The “CTR” refers to control cells that received none of the treatments. The treatments were: LPS10 and LPS50 = 10 and 50 µg/mL lipopolysaccharide (LPS) from *Escherichia coli*, LTA10 and LTA50 = 10 and 50 µg/mL lipoteichoic acid (LTA) from *Staphylococcus aureus*, ETxB-1 and -2 = 20 and 50 µg/mL subunit B of heat-labile enterotoxin of *Escherichia coli*, Flag-1 and -2 = 100 and 250 ng/mL flagellin from *Salmonella* Typhimurium, PMA-1 and -2 = 100 and 1000 ng/mL phorbol myristate acetate (PMA), poly-IC-1 and -2= 50 and 100 µg/mL polyinosinic polycytidylic acid (poly I:C). Asterisks over the boxes refer to significant differences compared to “CTR” cells within the same cell culture model and the same study. * *p* < 0.05.

IL-6 concentration of the cell-free supernatant was significantly decreased after 50 µg/mL LTA treatment (*p* = 0.0159) in 3D cultures (Figure 5C), but no significant effect was detected in 2D cultures (Figure 5A). The IL-6 concentration was significantly elevated in the culture media of 2D cultures after 250 ng/mL flagellin (*p* = 0.0195), 1000 ng/mL PMA (*p* = 0.0286) and 50 ng/mL poly I:C treatments (*p* = 0.0195) (Figure 5B), and following 100 ng/mL flagellin (*p* = 0.0357), 100 ng/mL PMA (*p* = 0.0498) and 50 ng/mL poly I:C exposures (*p* = 0.0358) in 3D cultures (Figure 5D).

### 3.4. IL-8 Concentration

**Figure 6:**
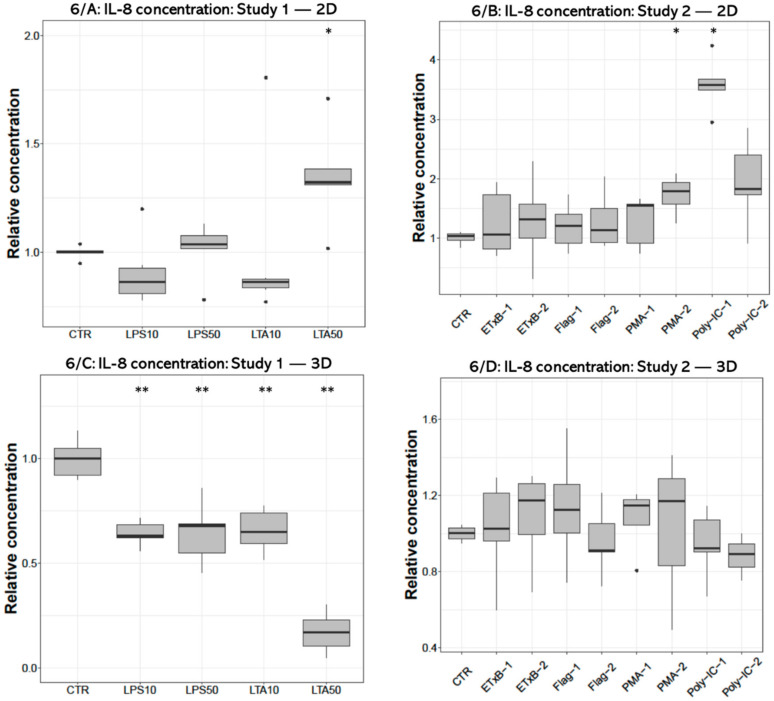
Boxplots showing the interleukin-8 (IL-8) concentration in cell culture media of hepatocyte—non-parenchymal cells in 2D (Study 1: (**A**) and Study 2: (**B**)) and 3D (Study 1: (**C**) and Study 2: (**D**)) co-cultures detected by a chicken specific ELISA assay (n_study 1_ = 6/group, n_study 2_ = 5/group). Relative concentrations were calculated by considering the mean value of control cultures as 1. The “CTR” refers to control cells that received none of the treatments. The treatments were: LPS10 and LPS50 = 10 and 50 µg/mL lipopolysaccharide (LPS) from *Escherichia coli*, LTA10 and LTA50 = 10 and 50 µg/mL lipoteichoic acid (LTA) from *Staphylococcus aureus*, ETxB-1 and -2 = 20 and 50 µg/mL subunit B of heat-labile enterotoxin of *Escherichia coli*, Flag-1 and -2 = 100 and 250 ng/mL flagellin from *Salmonella* Typhimurium, PMA-1 and -2 = 100 and 1000 ng/mL phorbol myristate acetate (PMA), poly-IC-1 and -2= 50 and 100 µg/mL polyinosinic polycytidylic acid (poly I:C). Asterisks over the boxes refer to significant differences compared to “CTR” cells within the same cell culture model and the same study. * *p* < 0.05, ** *p* < 0.01.

The concentrations of IL-8 were significantly increased by 50 µg/mL LTA treatment (*p* = 0.0133) in 2D cultures (Figure 6A), and decreased after applying all LPS (*p* = 0.008, *p* = 0.008, respectively) and LTA concentrations (*p* = 0.008, *p* = 0.008) in 3D cultures (Figure 6C). The 1000 ng/mL PMA (*p* = 0.0286) and 50 µg/mL poly I:C challenges significantly increased the concentration of IL-8 (*p* = 0.036) in 2D cultures (Figure 6B) but had no significant effect in 3D cultures (Figure 6D).

## 4. Discussion

In the present study, a 3D hepatocyte—non-parenchymal cell co-culture was successfully established utilizing a magnetic bioprinting method described by Desai et al. [31]. The applied hepatocyte and non-parenchymal cell fractions have been previously characterized by flow cytometry and immunofluorescent detection of specific markers for hepatocytes and non-parenchymal macrophages [24]. The 3D cell cultures comprised of these cell isolates developed adequately, successfully formed spheroids, and remained viable after three days of culturing as demonstrated by the measurement of metabolic activity and LDH leakage assay. Remarkable differences were observed between 2D and 3D hepatocyte—non-parenchymal cell co-cultures concerning their metabolic and inflammatory responses, corresponding to the findings of other authors [32,33,34,35]. The 3D cultures can provide a more proper model of cells growing in vivo in terms of gene expression, signaling pathways, molecular mechanisms and structure, because they can possess real cell-cell interactions. In contrast, 2D-cultured cells lose their natural polarity and topology due to limited cell-cell contacts on the adhesive surface of culture dishes. [36]. Moreover, organoid cell cultures retain the key features of specific diseases *in vitro*; meanwhile, they show long-term genetic stability and extended viability [37,38]. Further, these stem cell derived 3D cultures hold a promise for future tumor therapy research as the targeted culture readily shows the gene expression of the mimicked pathology even for months [39,40,41,42,43].

The main goal of our research was to create an inflammatory model on 2D and 3D hepatic cell cultures of chicken origin. There is only limited data available related to this issue; however, it would be an essential basis for future studies concerning the *in vitro* testing of anti-inflammatory agents. The applied 2D primary hepatocyte—non-parenchymal cell co-cultures of chicken origin have already been used in previous studies to investigate the cellular effects of acute heat stress and T-2 toxin [24,44]. Furthermore, even inflammatory models were designed using similar cultures of porcine origin [45]. The inclusion of the non-parenchymal cell fraction in co-cultures at cell ratio 6:1 (hepatocytes to non-parenchymal cells) refers to a mild hepatic inflammatory state with moderate intrahepatic macrophage migration [24], enabling the investigation of the link between the inflammatory and stress response. However, the differences between 2D and 3D chicken hepatocyte—non-parenchymal cell co-cultures as inflammatory models have not yet been revealed.

In the first part of the present research (Study 1) we intended to examine the effects of bacterial endotoxins (LPS of Gram-negative and LTA of Gram-positive origin) as traditional proinflammatory agents. As the applied endotoxin treatments except LTA applied in 50 µg/mL could not induce proinflammatory cytokine production in the cell cultures used, further potential PAMPs (ETxB from *Escherichia coli*, flagellin from *Salmonella* Typhimurium, PMA and poly I:C) were screened in Study 2.

At first, it was aimed to monitor how the applied proinflammatory agents affected the metabolic activity and membrane damage of the cultured cells. Elevations of metabolic activity, measured with CCK-8 test were observed in 3D cell cultures after treatment with both concentrations of LPS and with 50 µg/mL LTA, but no changes were detected in 2D cultures. These elevations indicate that the cells tried to adapt to the bacterial endotoxins as a compensatory mechanism and turned into a more active metabolic state. Apparent differences could be seen between 2D and 3D cultures, suggesting the increased sensitivity of 3D cultured cells to these PAMPs. Furthermore, these results indicate a faster and more efficient hepatocellular metabolic adaptation to environmental impacts than in the case of 2D cultures. Similarly, it has been proven in a previous study that 3D-cultured cells can be more adaptive to cytotoxic agents (such as H_2_O_2_, methotrexate or neratinib) than 2D cultures [46,47,48].

On the contrary, ETxB treatment elevated the metabolic activity of the 2D cultures, but had no significant effect on the 3D-cultured cells, which indicates that 2D cells were more responsive to this agent. PMA and poly I:C treatment decreased the metabolic activity of the 2D cell cultures, demonstrating a metabolically depressed state because of the harmful effect of these compounds. However, cell membrane damage was only detectable after the poly I:C treatment based on the extracellular LDH measurements.

Autophagy plays an essential role in the degradation and recycling of irregular or malfunctional cellular materials and organelles and maintaining the homeostasis in the liver [49] and other organs; therefore, it has a principal role in cytoprotection. For example, it has been proven that autophagy is a cytoprotective response to LPS-induced cardiomyocyte injury [50]. In the 3D co-cultures of the present study, extracellular LDH activities decreased when treated with LPS and LTA compared to the control group. It could indicate an increase in the autophagocytosis of the hepatocytes and the Kupffer cells, possessing a cytoprotective effect as injured organelles are getting non-selectively sequestered [51], leading to decreased LDH release. These results are supported by the previous finding of Kundu et al. [52], where high concentrations of LPS treatment resulted in decreased LDH release in prostate epithelial cells, and also in the study of Li et al. [53], where they proved that low-dose LPS has neuroprotective effects.

The proinflammatory effects of our candidate molecules were screened by measuring the concentrations of IL-6 and IL-8 in culture media. Notwithstanding that some other pro- and anti-inflammatory mediators should be investigated in further studies, and the finite number of the screened cytokines is a limitation of the present study, monitoring the hepatocellular IL-6 and IL-8 response provided sufficient initial data concerning the proinflammatory action of the tested candidates. Significant IL-8 elevation was detected after the higher dosage LTA treatment in 2D cultures, although in accordance with the hypotheses of cellular compensation and autophagy, the same treatment yielded significantly decreased IL-6 and IL-8 concentrations in 3D conditions. Interestingly, a dose-dependent selective inflammatory activation of 2D and 3D cultures were observed in Study 2: significant elevation of IL-6 level ensued the 100 ng/mL PMA and flagellin treatments in 3D, meanwhile, the higher dosages of these agents proved to be ineffective in the same 3D model, yet they provoked a significant surge of at least one of the proinflammatory cytokines in 2D cultures. This phenomenon is possibly the result of the aforementioned differences of the cultures both in sensitivity and compensatory mechanisms. Spheroids might react to lower dosages of the same stimuli but possibly activate over-compensatory anti-inflammatory mechanisms when subjected to detrimental PAMP concentration.

The lower-dose, 50 µg/mL poly I:C supplementation turned out to be the most potent and versatile proinflammatory treatment as it has triggered the increase of both cytokines in 2D and similarly had a significant impact on the IL-6 level in 3D cultures. This novel molecule showed a potent effect in splenocyte-derived leukocyte and ovi-duct originated primary chicken cell cultures inducing high IL-1 beta IL-6, IFN-alpha, and beta mRNA level elevation [29,30,54]. Kim et al. described a TLR signal independent route in which PKC signal transduction can enhance IL-6 production. This mechanism is related to a cytoskeletal regulatory protein and actin bundling which is essential for the translation of IL-6 mRNA. In accordance with our results, PMA as a direct activator of PKC would serve as potent agent to activate independent and separate steps of TLR mediated cytokine production in the absence of a PAMP both in 2D- and 3D-cultured cells [18,19,55].

Neither of the applied proinflammatory treatments provoked elevated IL-8 secretion in 3D cultures, moreover, each applied endotoxin treatment resulted in a significant reduction of IL-8 protein level in Study 1. Therefore, according to the present study, the level of this interleukin may not be optimal to characterize inflammatory response in 3D chicken hepatic models. It can be stated that LPS and ETxB had a proinflammatory effect neither in 2D nor in 3D cultures. The inadequate IL response caused by *Escherichia coli* LPS is a possible result of the deficient signal transduction of chicken TLR4.

TLRs specified for extracellular pathogen recognition exclusively activate myeloid differentiation primary response protein 88 (MyD88) dependent pathway which is needed for the prompt activation of Nf-κB. In human TLR-3 and -4 adaptors, double stranded RNA and LPS can set off interferon production and a so-called delayed or late-phase activation of NF-kappa B via a sovereign MyD88 independent route. The inadequate IL response caused by Escherichia coli LPS is possibly related to the deficient signal transduction of chicken TLR4 and the lack of this additional route. This hypothesis was confirmed by Keestra and van Putten by the cited lack of interferon production in response to different *Salmonella* Enteritidis and Gallinarum as well as *Pasteurella multocida* and secondly by the absence of certain TLR4 signal mammalian gene orthologs in chicken [6,9,10,55,56]. This concept is still controversial as two different research groups managed to trigger INF response with *Escherichia coli* and *Salmonella* Typhimurium in cell cultures of chicken origin [56,57]; however, only on the level of gene expression. It can be hereby stated that IL-6 and IL-8 protein level elevation could not be induced in cell culture media with LPS from the chicken pathogenic O55:B5 serotype of *E. coli* in accordance with the relatively high tolerance to LPS in avian species [58]. Nonetheless, there is just a handful of published data on the effect of LPS on the secreted IL-6 and IL-8 concentrations in chicken. These interleukin levels showed only a slight or no increase as opposed to the remarkable elevation of the respective gene expressions detected by the RT-PCR method [29,30,59,60]. As both interleukins were measured directly from cell culture media, the differences between the present results and those of studies assessing mRNA levels might arise from post-transcriptional, translational and post-translational regulatory mechanisms. These processes, for example interleukin mRNA accumulation or degradation by ribonuclease enzymes and PKC dependent translational regulation of IL-6 level are included in the determination of the final inflammatory cytokine response of the cell, especially under 3D conditions [34,55,61].

The results of the present study show that there are major differences in the inflammatory responses between 2D and 3D hepatic cell models, which can be supported by numerous hypotheses. It has been proved that 3D cultures produce less proinflammatory interleukins (e.g., IL-6 and IL-8) than 2D cultures from the same cell type [33]. One explanation may be that 3D cell cultures are more likely to adapt to stress factors, and they might be able to protect themselves more efficiently under harmful conditions. Cell-to-cell and cell-to-environment connections also have a significant impact on cell reactivity and viability, and many differences have been detected comparing 2D and 3D cell culture types in this regard. For example, it has been proved that 3D cultures have lower mRNA and protein levels of actin, and when the actin polymerization has been inhibited by mycalolid-B in 3D cell cultures, the cell-to-cell connections decreased resulting in elevated IL-6 secretion [34]. It seems, based on these findings, that the more stable the cell-to-cell contacts are, the more capable the cell culture is to protect itself from detrimental inflammatory effects. It was also found that 3D human mesenchymal stem cell cultures have lower cytoskeletal tension compared with 2D ones, which has been associated with morphological and mechanical changes [35]. Another explanation may arise from the higher anti-inflammatory mediator production rate in the 3D cultures [32], and therefore they are more likely to alleviate the inflammatory response.

Genetic and epigenetic factors can also play a considerable role in regulating inflammatory mechanisms. Seno et al. [34] exposed many differences between the transcriptomic profile of 3D-, and 2D-cultured cells, especially regarding inflammation-related molecules with RNA-seq transcriptome analysis. According to their results, IL-6 mRNA levels in 3D-cultured cells were higher, but the protein secretion levels were lower than in 2D-cultured ones. They also suggested certain post-transcriptional modification differences of the IL-6 mRNA, because the level of regnase-1 enzyme, the regulatory RNase of inflammatory cytokines was increased in 3D cultures. It has been assumed too, that the genetic profile of hepatocytes cultured in 2D conditions is hardly comparable to the in vivo growing cells, but 3D-cultured hepatocytes, often called hepatospheres, could be much closer to the living state. For example, when the gene expression of four different culture conditions, including different monolayers and 3D cultures have been opposed, from the 242 liver-specific genes that have been screened, 85% were stably expressed in hepatospheres cultured with the rocked technique [5]. Other studies also suggested that there are many differences between the genetic profile of hepatocytes cultured with 3D and 2D techniques. For example, it has been proved that in hepatospheres, genes involved in xenobiotic and lipid metabolism were expressed more robustly compared to their expression in 2D cultures [62].

## 5. Conclusions

In conclusion, 3D spheroids of hepatocyte—non-parenchymal cell co-cultures have been successfully established with magnetic bioprinting from primarily isolated hepatic cell fractions of chickens. Based on our results, both 2D and 3D co-cultures can serve as proper models for *in vitro* investigations of the inflammatory and stress response of the avian liver. However, the different response of 2D and 3D cell cultures to the applied potential proinflammatory agents should be carefully addressed as 2D cultured cells were more responsive to the cited uppermost concentrations (2, 5–10 times elevated) of LTA, flagellin and PMA, while low-set dosages mostly influenced 3D cultures. Bacterial endotoxins could remarkably stimulate the metabolic activity of 3D cultured cells without enhancing the production of the investigated proinflammatory cytokines; in contrast, the IL-8 release of 3D cultures was decreased by all LPS and LTA treatments, suggesting the effective metabolic adaptation and the presumably initiated anti-inflammatory mechanisms of the spheroids. The viral RNA analogue poly I:C caused a moderate metabolic depression on 2D cultures coupled to partial cellular damage and significant elevation of IL-6 levels on both 2D and 3D and increased IL-8 production on 2D cultures. Summarizing these results, the applied avian hepatic cell models seemed to be relatively resistant to the studied LPS and ETxB, possibly due to the observed high metabolic adaptation potential and the aforementioned specialties of avian inflammatory signal pathways contributing to a better tolerance to LPS. The present study provided novel data on the hepatic inflammatory homeostasis by screening a wide range of potential proinflammatory agents and highlighted the difficulties of activating hepatic innate immunity in chicken, which is a key finding to study the inflammatory and stress response in the avian liver.

## Data Availability

All raw data supporting the results of the present study can be obtained from the corresponding author upon reasonable request.
